# *Lactobacillus paracasei* KW3110 Suppresses Inflammatory Stress-Induced Premature Cellular Senescence of Human Retinal Pigment Epithelium Cells and Reduces Ocular Disorders in Healthy Humans

**DOI:** 10.3390/ijms21145091

**Published:** 2020-07-18

**Authors:** Takahiro Yamazaki, Hiroaki Suzuki, Sayuri Yamada, Konomi Ohshio, Miho Sugamata, Takahiro Yamada, Yuji Morita

**Affiliations:** 1 KIRIN Central Research Institute, Kirin Holdings Co., Ltd., Kanagawa 236-0004, Japan; Hiroaki_Suzuki@kirin.co.jp (H.S.); Sayuri_Yamada@kirin.co.jp (S.Y.); Konomi_Ohshio@kirin.co.jp (K.O.); sgmtmh@gmail.com (M.S.); Yuji_Morita@kirin.co.jp (Y.M.); 2 Ario Nishi-Arai Eye Clinic, Tokyo 123-0843, Japan; dr_yamada@orthomedico.jp

**Keywords:** lactic acid bacteria, probiotics, inflammation, cellular senescence, retina, eye fatigue

## Abstract

*Lactobacillus paracasei* KW3110 (KW3110) has anti-inflammatory effects and mitigates retinal pigment epithelium (RPE) cell damage caused by blue-light exposure. We investigated whether KW3110 suppresses chronic inflammatory stress-induced RPE cell damage by modulating immune cell activity and whether it improves ocular disorders in healthy humans. First, we showed that KW3110 treatment of mouse macrophages (J774A.1) produced significantly higher levels of interleukin-10 as compared with other lactic acid bacterium strains (all *p* < 0.01). Transferring supernatant from KW3110- and *E. coli* 0111:B4 strain and adenosine 5′-triphosphate (LPS/ATP)-stimulated J774A.1 cells to human retinal pigment epithelium (ARPE-19) cells suppressed senescence-associated phenotypes, including proliferation arrest, abnormal appearance, cell cycle arrest, and upregulation of cytokines, and also suppressed expression of tight junction molecule claudin-1. A randomized, double-blind, placebo-controlled parallel-group study of healthy subjects (*n* = 88; 35 to below 50 years) ingesting placebo or KW3110-containing supplements for 8 weeks showed that changes in critical flicker frequency, an indicator of eye fatigue, from the week-0 value were significantly larger in the KW3110 group at weeks 4 (*p* = 0.040) and 8 (*p* = 0.036). These results suggest that KW3110 protects ARPE-19 cells against premature senescence and aberrant expression of tight junction molecules caused by chronic inflammatory stress, and may improve chronic eye disorders including eye fatigue.

## 1. Introduction

A variety of living organisms have an evolved immune system to fight off infections or other stresses. Inflammation is an important biological response to eliminate infectious factors and damaged cells and tissues, and then initiate tissue repair. It is well established that chronic inflammatory responses are induced by various daily stresses including aging and mental stress, as well as bacterial and viral infections, leading to different disorders or diseases [1,2,3,4]. Thus, preventive and/or therapeutic strategies are required to suppress the conditions related to those stresses.

To prevent disorders induced by chronic inflammation, several food materials with anti-inflammatory effects have been studied. For example, lactic acid bacteria (LAB) are well known to enhance gut barrier function and improve the immune system, and are used as probiotics and paraprobiotics [5]. We previously reported that a specific strain of LAB, *Lactobacillus paracasei* KW3110 (KW3110), has anti-allergic and anti-inflammatory effects in mice and humans [6,7,8,9].

KW3110 has also been shown to protect retinal pigment epithelium (RPE) cells from blue-light exposure in mice and to reduce visual display terminal-induced ocular disorders in humans [10,11]. As well as those types of stress, long-term exposure to oxidants as a chronic stress induces RPE cell damage [12]. However, ocular disorders develop not only from direct and chronic stress to the retina, but also from stress signals from remote tissues. For example, increased systemic levels of pro-inflammatory cytokines may be involved in age-related macular degeneration, and mental stress caused by continual calculation work, which is simple and boring but requires continued precision and vigilance, might induce ocular disorders [13,14,15,16]. However, the preventive effects and mechanisms of KW3110 on systemic and chronic stress-induced ocular disorders remain unknown.

In this study, we investigated whether KW3110 suppresses RPE cell damage induced by chronic inflammatory stress signaling of immune cells using macrophages and RPE cells. In addition, we performed a randomized, double-blind, placebo-controlled parallel-group study of 88 healthy humans for 8 weeks to evaluate the effects of KW3110 on chronic ocular disorders. Lastly, the effects of KW3110 on mental stress-induced ocular disorders were examined.

## 2. Results

### 2.1. In Vitro Experiments

#### 2.1.1. KW3110 Has a Stronger Anti-Inflammatory Effect than LAB from Commercial Products

**Figure 1:**
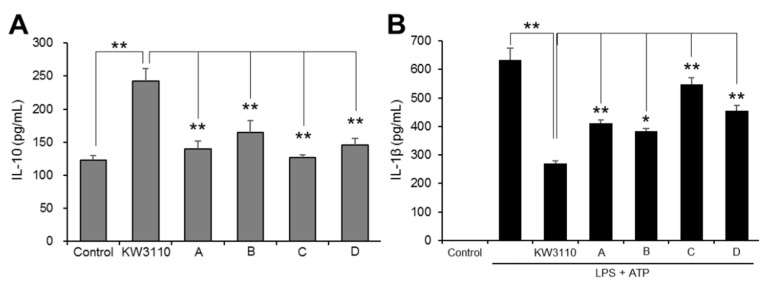
Comparative analysis of the effects of lactic acid bacteria (LAB) on cytokine production in J774A.1 cells. (**A**) J774A.1 cells were treated with KW3110 or LAB strains A–D (5 μg/mL) for 24 h and IL-10 in the supernatant was measured by enzyme-linked immunosorbent assay (ELISA). (**B**) J774A.1 cells were treated first with KW3110 or LAB strains A–D (5 μg/mL) for 24 h, and then with LPS (10 μg/mL) for 4 h and ATP (2 mM) for 1 h. Interleukin (IL)-1β in the supernatant was measured by ELISA. Values indicate means ± SEM (*n* = 3). Statistical differences were analyzed by analysis of variance (ANOVA) followed by Tukey’s test (*, *p* < 0.05; **, *p* < 0.01).

KW3110 induces the production of interleukin-10 (IL-10) in macrophages [10,11], but the impact of KW3110 on IL-10 production relative to other LAB is not yet clear. We therefore compared the effects of KW3110 and other LAB strains from commercial products on the induction of IL-10 in J774A.1 cells, which are known to produce IL-10 [17]. KW3110 treatment of J774A.1 cells significantly increased IL-10 levels as compared with control treatment (*p* < 0.001), whereas IL-10 levels in cells treated with all other LAB were significantly lower as compared with KW3110-treated cells (A, *p* = 0.001; B, *p* = 0.010; C, *p* < 0.001; D, *p* = 0.002; Figure 1A).

Because IL-10 suppresses the expression of pro-inflammatory cytokine IL-1β in activated macrophages [18,19], we investigated whether KW3110 suppresses IL-1β production in J774A.1 cells and compared the effects of KW3110 with those of other LAB. A high level of IL-1β was detected in ultrapure lipopolysaccharide from *E. coli* 0111:B4 strain and adenosine 5′-triphosphate (LPS/ATP)-stimulated J774A.1 cells, but KW3110 treatment of these cells significantly suppressed IL-1β levels (*p* < 0.001; Figure 1B). By contrast, IL-1β levels in cells treated with all other LAB were significantly higher as compared with KW3110-treated cells (A, *p* = 0.004; B, *p* = 0.023; C, *p* < 0.001; D, *p* < 0.001; Figure 1B).

#### 2.1.2. ARPE-19 Cell Proliferation, But Not Cell Death, Is Affected by KW3110 or LPS/ATP Stimulation of Macrophages

**Figure 2:**
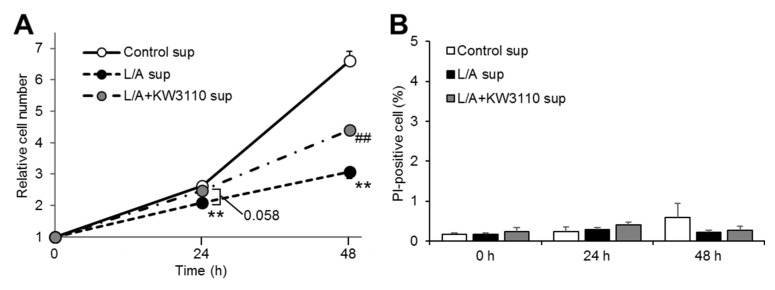
Effects of the supernatant from *Lactobacillus paracasei* KW3110 (KW3110)- and *E. coli* 0111:B4 strain and adenosine 5′-triphosphate (LPS/ATP)-stimulated J774A.1 cells on ARPE-19 cell proliferation and cell death. ARPE-19 cells incubated for 0, 24, or 48 h after supernatant transfer were stained with Hoechst 33342 (1 μg/mL) and PI (1 μg/mL) solutions. (**A**) Number of Hoechst 33342-positive cells at 0, 24, or 48 h relative to 0 h was calculated. (**B**) PI-positive cells as a percentage of Hoechst 33342-positive cells at 0, 24, or 48 h was calculated. Control sup, cells exposed to supernatant from non-stimulated J774A.1 cells; L/A sup, cells exposed to supernatant from LPS/ATP-stimulated J774A.1 cells; L/A + KW3110 sup, cells exposed to supernatant from KW3110- and LPS/ATP-stimulated J774A.1 cells. Values indicate means ± SEM (*n* = 6–12). Statistical differences were analyzed by ANOVA followed by Tukey’s test (**, *p* < 0.01 vs. Control sup; ^##^, *p* < 0.01 vs. L/A sup).

To investigate whether LPS/ATP-stimulated macrophages induce RPE cell stress and the effect of KW3110 on this stress, we evaluated the effects of supernatant from KW3110-stimulated J774A.1 cells on cell proliferation and cell death in ARPE-19 cells. The relative cell number was significantly lower in the LPS/ATP-stimulated J774A.1 cell supernatant transfer group (L/A sup) than in the non-stimulated J774A.1 cell supernatant transfer group (Control sup) at both 24 and 48 h after supernatant transfer (24 h, *p* = 0.007; 48 h, *p* < 0.001; Figure 2A). The relative cell number in the KW3110- and LPS/ATP-stimulated J774A.1 cell supernatant transfer group (L/A + KW3110 sup) tended to be higher at 24 h and was significantly higher at 48 h after supernatant transfer as compared with L/A sup (24 h, *p* = 0.058; 48 h, *p* = 0.002; Figure 2A). By contrast, the percentage of propidium iodide (PI)-positive cells was not different among the three groups at any time point (Figure 2B).

#### 2.1.3. Effects of KW3110 on Prematurely Senescent Phenotypes of Inflammatory-Stressed ARPE-19 Cells

**Figure 3:**
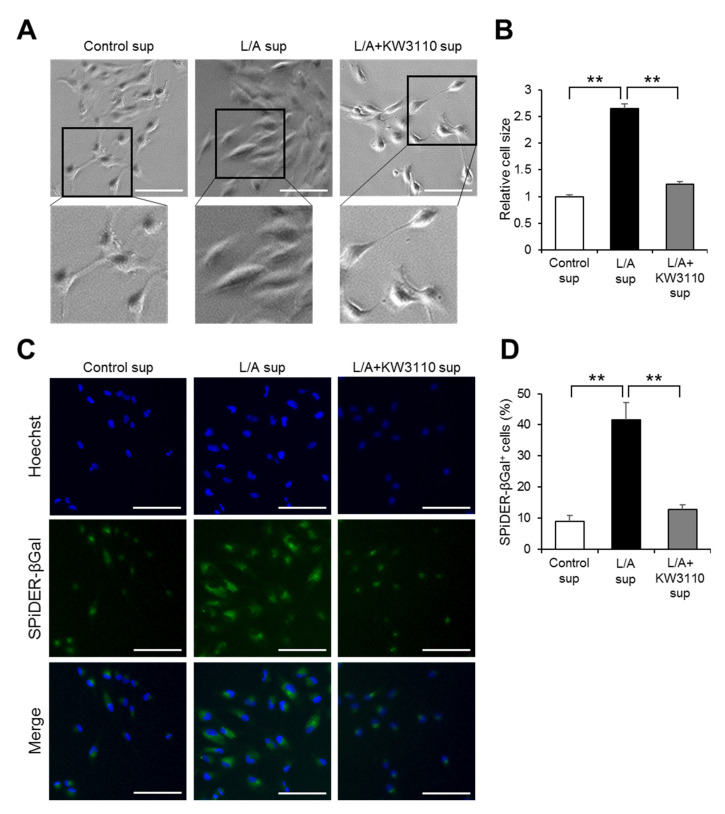
Analysis of the prematurely senescent phenotype of ARPE-19 cells exposed to supernatant from KW3110- and LPS/ATP-stimulated J774A.1 cells. ARPE-19 cells incubated for 24 h after supernatant transfer were stained with SPiDER-βGal solution containing Hoechst 33342, and bright-field image analyses (**A**), cell size comparisons (**B**), fluorescence image analyses for Hoechst 33342 and SPiDER-βGal (**C**), and comparisons of ratio of SPiDER-βGal-positive cells to Hoechst 33342-positive cells (**D**) were performed. Control sup, cells exposed to supernatant from non-stimulated J774A.1 cells; L/A sup, cells exposed to supernatant from LPS/ATP-stimulated J774A.1 cells; L/A + KW3110 sup, cells exposed to supernatant from KW3110- and LPS/ATP-stimulated J774A.1 cells. Scale bar, 100 μm. Values indicate means ± SEM (*n* = 3). Statistical differences were analyzed by ANOVA followed by Tukey’s test (**, *p* < 0.01).

The impairment of cell proliferation ability, as observed in Figure 2, is a representative phenotype of premature cellular senescence [20]. We therefore investigated whether the premature senescence of RPE cells is induced by LPS/ATP-stimulated macrophages and rescued by KW3110. Bright-field images showed that ARPE-19 cells became flat and enlarged in L/A sup as compared with Control sup (Figure 3A), whose cellular phenotype was characterized as prematurely senescent [20]. The appearance of the cells in L/A + KW3110 sup was same as that in Control sup (Figure 3A). Quantitatively, the cell size in L/A sup was significantly larger as compared with Control sup (*p* = 0.003; Figure 3B), while this enlargement was significantly suppressed in L/A + KW3110 sup (*p* = 0.004; Figure 3B). Furthermore, we performed staining with the senescent cell marker SPiDER-βGal, which showed enhanced signals and significant increase of SPiDER-βGal-positive cell ratios in L/A sup as compared with Control sup (Figure 3C) (*p* = 0.002; Figure 3D). This effect was significantly suppressed in L/A + KW3110 sup as compared with L/A sup (Figure 3C) (*p* = 0.003; Figure 3D).

#### 2.1.4. Effects of KW3110 on Cell Cycle Arrest and Senescence-Associated Secretory Phenotype (SASP)-Related Gene Upregulation in Inflammatory-Stressed ARPE-19 Cells

**Figure 4:**
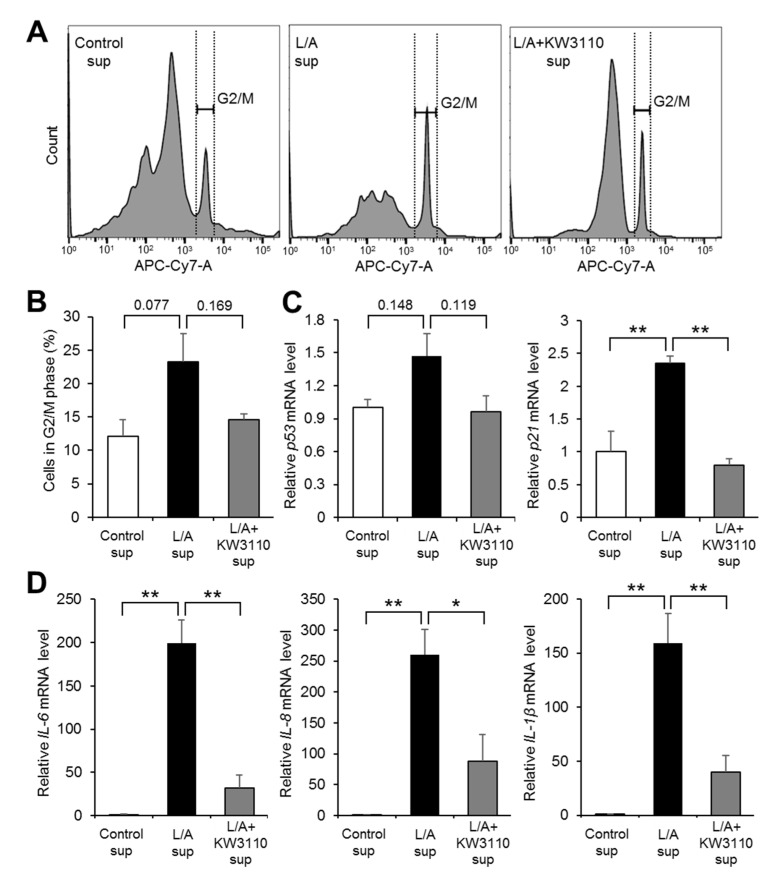
Evaluation of cell cycle arrest and senescence-associated secretory phenotype (SASP)-related gene expression in ARPE-19 cells exposed to supernatant from KW3110- and LPS/ATP-stimulated J774A.1 cells. (**A**,**B**) ARPE-19 cells incubated for 24 h after supernatant transfer were stained with Cell Cycle Assay Solution Deep Red. Representative flow cytometry charts (A) and the ratio of G2/M phase cells to total cells (B) are shown. (**C**,**D**) Total RNA from ARPE-19 cells incubated for 24 h after supernatant transfer was extracted and reverse-transcribed. Quantitative RT-PCR was performed to amplify human *GAPDH*, *p53*, *p21*, *IL-6*, *IL-8*, and *IL-1β*. All values were normalized to the expression of *GAPDH*. Control sup, cells exposed to supernatant from non-stimulated J774A.1 cells; L/A sup, cells exposed to supernatant from LPS/ATP-stimulated J774A.1 cells; L/A + KW3110 sup, cells exposed to supernatant from KW3110- and LPS/ATP-stimulated J774A.1 cells. Values indicate means ± SEM (*n* = 3). Statistical differences were analyzed by ANOVA followed by Tukey’s test (*, *p* < 0.05; **, *p* < 0.01).

Because prematurely senescent cells are arrested in the G2/M phase [21], we investigated whether inflammatory signals derived from LPS/ATP-stimulated macrophages induce cell cycle arrest in RPE cells and the effect of KW3110 on this arrest. The ratio of G2/M phase cells to total cells tended to be higher in L/A sup than in Control sup (*p* = 0.077; Figure 4A,B). The increased ratio in L/A sup was partially suppressed in L/A + KW3110 sup (*p* = 0.169; Figure 4A,B). Expression levels of two cell cycle arrest-related genes (*p53* and *p21*) were upregulated in L/A sup as compared with Control sup (*p53*, *p* = 0.148; *p21*, *p* = 0.007; Figure 4C), and this gene upregulation in L/A sup was suppressed in L/A + KW3110 sup (*p53*, *p* = 0.119; *p21*, *p* = 0.004; Figure 4C).

We also investigated whether inflammatory signals derived from LPS/ATP-stimulated macrophages induce SASP, including upregulation of pro-inflammatory cytokines, in RPE cells and whether KW3110 can rescue cells from these phenotypes. The expression levels of three representative SASP-related genes, *IL-6*, *IL-8*, and *IL-1β*, were significantly upregulated in L/A sup as compared with Control sup (*IL-6*, *p* < 0.001; *IL-8*, *p* = 0.004; *IL-1β*, *p* = 0.002; Figure 4D), and this gene upregulation in L/A sup was significantly suppressed in L/A + KW3110 sup (*IL-6*, *p* = 0.001; *IL-8*, *p* = 0.029; *IL-1β*, *p* = 0.008; Figure 4D).

#### 2.1.5. Effects of KW3110 on the Expression of Tight Junction Molecules in Inflammatory-Stressed ARPE-19 Cells

**Figure 5:**
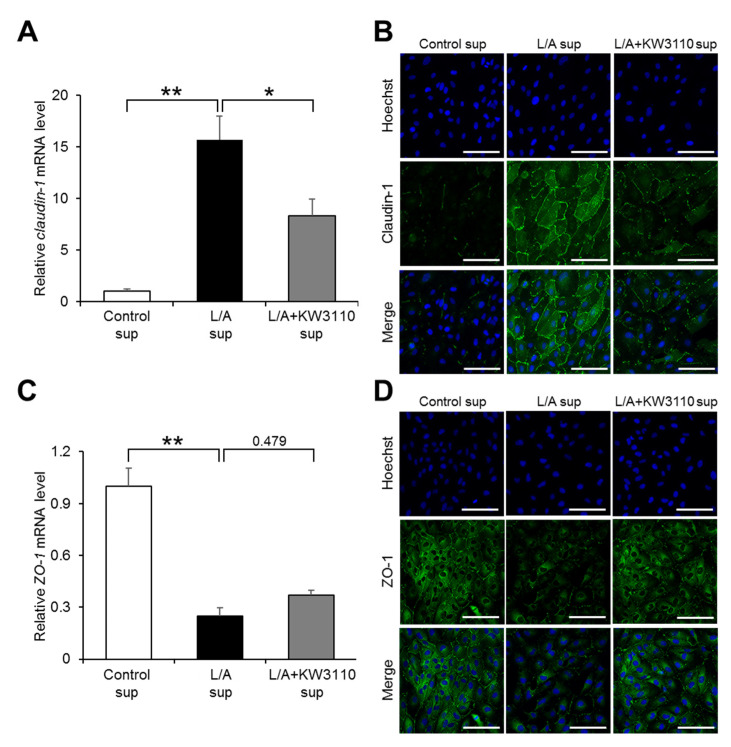
Expression of tight junction molecules in ARPE-19 cells exposed to supernatant from KW3110- and LPS/ATP-stimulated J774A.1 cells. (**A**,**C**) Total RNA from ARPE-19 cells incubated for 24 h after supernatant transfer was extracted and reverse-transcribed. Quantitative RT-PCR was performed to amplify human *GAPDH*, *claudin-1*, and *zonula occludens-1* (*ZO-1)*. Values were normalized to the expression of *GAPDH* and expressed as means ± SEM (*n* = 3). Statistical differences were analyzed by ANOVA followed by Tukey’s test (*, *p* < 0.05; **, *p* < 0.01). (**B**,**D**) ARPE-19 cells incubated for 24 h after supernatant transfer were stained with anti-Claudin-1 or anti-ZO-1 antibody and Hoechst 33342, and fluorescence images for Hoechst 33342, claudin-1, or ZO-1 staining were obtained. Control sup, cells exposed to supernatant from non-stimulated J774A.1 cells; L/A sup, cells exposed to supernatant from LPS/ATP-stimulated J774A.1 cells; L/A + KW3110 sup, cells exposed to supernatant from KW3110- and LPS/ATP-stimulated J774A.1 cells. Scale bar, 100 μm.

RPE cells form an important permeability barrier, which is supported by tight junctions between the cells [22]. We investigated whether inflammatory signals derived from LPS/ATP-stimulated macrophages affect the expression of tight junction molecules. Expression levels of the *claudin-1* and *zonula occludens-1* (*ZO-1*) genes, encoding integral membrane components that form tight junctions, were significantly upregulated and downregulated, respectively, in L/A sup as compared with Control sup (*claudin-1*, *p* = 0.002; *ZO-1*, *p* < 0.001; Figure 5A,C). The change in *claudin-1* gene expression level in L/A sup was partially but significantly reversed in L/A + KW3110 sup (*p* = 0.041; Figure 5A); however, that in *ZO-1* gene expression level was not affected in L/A + KW3110 sup (*p* = 0.479; Figure 5C). Although the effects of KW3110 on the expression levels of these genes were mild, immunostaining showed that the signals reflecting claudin-1 or ZO-1 protein expression increased or decreased, respectively, in L/A sup as compared with Control sup, and the change was reversed in L/A + KW3110 sup as compared with L/A sup (Figure 5B,D).

### 2.2. Clinical Study

#### 2.2.1. Background Information and Baseline Characteristics of the Subjects

**Figure 6:**
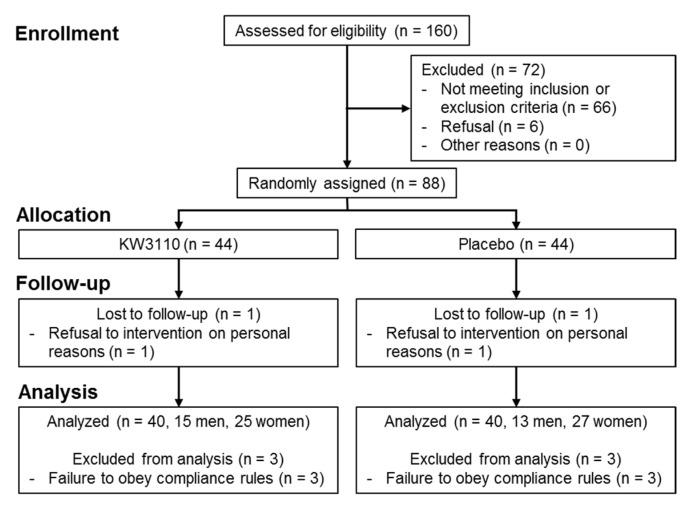
Flow diagram showing recruitment of the study subjects and data analysis.

Figure 6 shows the flow chart of participant recruitment and data analysis in the clinical study. Overall, 72 of 160 participants attending the screening test were excluded in accordance with the inclusion and exclusion criteria; as a result, 88 subjects (30 male, 58 female) were judged eligible by the supervising physician, and were randomly assigned to the placebo or KW3110 group (*n* = 44 per group). Two subjects subsequently refused the intervention owing to personal reasons. In accordance with the exclusion criteria, of the 86 subjects who finished the study (*n* = 43 per group), six were excluded from primary and secondary endpoint evaluation by the supervising physician before the blinding was revealed because they did not follow the compliance rules.

Consequently, data analyses for primary and secondary endpoint evaluation were performed on 80 subjects (*n* = 40 per group). The baseline characteristics of these 80 subjects measured at the screening test are summarized in Table 1. There were no significant differences in any parameters between the two groups.

#### 2.2.2. Primary Outcomes

**Figure 7:**
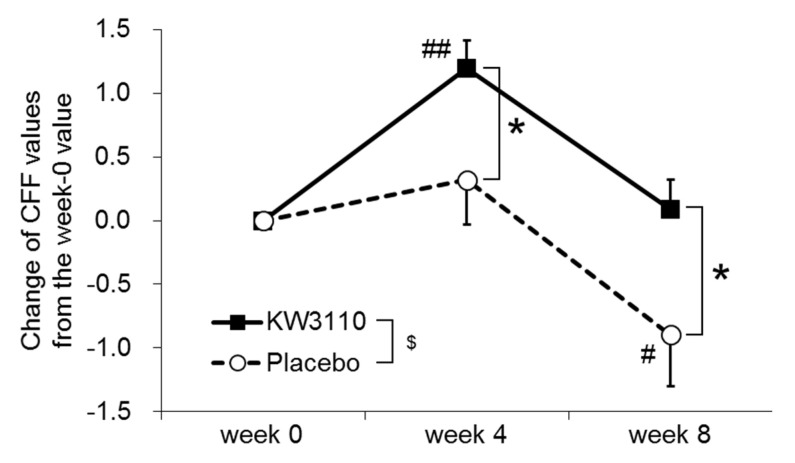
Effects of KW3110 on changes in CFF value from week 0. Data are calculated as the degree in change from the week-0 value, and expressed as means ± SEM. Comparisons of change in CFF value from the week-0 value between the two groups were performed by two-way repeated measures ANOVA followed by LSD test (^$^, *p* < 0.05; main effect of ingestion) (*, *p* < 0.05; effect of ingestion at each time point). Within-group comparisons between week 0 and week 4 or week 8 were performed by repeated measures ANOVA followed by LSD test (^#^, *p* < 0.05; ^##^, *p* < 0.01).

Table 2 summarizes the results of the critical flicker frequency (CFF) tests before the Uchida–Kraepelin workload as an index of daily eye fatigue. The CFF value was significantly increased at week 4 relative to week 0 in the KW3110 group, but was significantly decreased at week 8 relative to week 0 in the placebo group. The changes in CFF from week 0 to weeks 4 and 8 were significantly larger in the KW3110 group than in the placebo group (Figure 7). Changes in the criteria “eye redness” and “unfocused vision” on the visual analogue scale (VAS) from week 0 to weeks 4 and 8 were significantly larger in the KW3110 group than in the placebo group (Table S1). No significant differences in other primary outcomes at any time points were detected between the two groups (Tables S1–S3).

To evaluate mental stress-induced ocular disorders, the influence of the Uchida–Kraepelin workload on CFF values, high frequency component (HFC-1) values, and miosis rates were evaluated (Table S4). The change in CFF values measured before and after the workload at week 0 differed significantly between the two groups. The miosis rates after the workload and the changes between the rates measured before and after the workload at week 0 differed significantly between the two groups; in addition, the miosis rates increased after workload as compared with the before-workload values at week 0. These results suggest that the Uchida–Kraepelin test did not induce an ocular disorder related to mental stress, at least in this study. As a result, the supervising physician decided that only values measured before the workload for all outcomes would be used as indices of chronic ocular disorder.

#### 2.2.3. Secondary Outcomes

The results of the VAS for fatigue, profile of mood states second edition (POMS2), and Uchida–Kraepelin tests are summarized in Tables S5–S7. In the POMS2 test, the “depression-dejection (DD)” value was significantly lower at week 4 in the KW3110 group than in the placebo group. No significant differences in other secondary outcomes at any time points were detected between the two groups (Tables S5–S7).

#### 2.2.4. Safety Endpoints

Safety endpoints were evaluated for 86 subjects who ingested the test supplements at least once, and the results are summarized in Tables S8–S12. The circulatory analysis showed that pulse rate before the ingestion period was significantly lower in the KW3110 group than in the placebo group (Table S8). The peripheral blood analysis showed that uric acid before the ingestion period was significantly higher in the KW3110 group than in the placebo group, while basophils and total protein significantly increased after the ingestion period relative to levels before the ingestion period in the KW3110 group (Table S10). The ophthalmic analysis showed that the intraocular pressure (average of both eyes, non-dominant eye, and right eye) at week 4 was significantly lower in the KW3110 group than in the placebo group, and the intraocular pressure (left eye) significantly increased at week 8 relative to the screening value in the placebo group (Table S11). No significant differences in other safety endpoints were detected between the two groups (Tables S8–S12). No adverse events related to the test supplement were reported throughout the study.

## 3. Discussion

Our study demonstrated that a specific strain of LAB, KW3110, induced IL-10 production and inhibited IL-1β production in macrophages to a greater extent than other LAB tested in the study. We also showed that KW3110 suppressed human RPE cell senescence and the aberrant expression of tight junction molecules induced by chronic inflammatory stress signals from macrophages. Furthermore, in a randomized, double-blind, placebo-controlled parallel-group study of healthy humans, KW3110 ingestion was found to improve the objective parameter of eye fatigue and the subjective parameter of depression.

KW3110 activates macrophages through phagocytosis and promotes the production of cytokines such as IL-10, leading to suppress IL-1β production through IL-10R signaling in macrophages [9,10,11,23]. Consistent with those results, in the present study, KW3110 induced the production of IL-10 in mouse and human immune cells to a greater extent than the other LAB tested in this study (Figure 1A and Figure S1). Moreover, KW3110 had a stronger effect on the suppression of IL-1β production in LPS/ATP-stimulated macrophages as compared with the other LAB strains (Figure 1B). Microbes including LAB are detected and phagocytosed by immune cells such as macrophages, which then upregulate the production of pro- or anti-inflammatory cytokines [24,25]. KW3110 may be more susceptible to phagocytosis by macrophages as compared with other LAB, resulting in greater induction of IL-10. Further investigation is needed to clarify whether the phagocytosis of KW3110 by macrophages is required for the production of IL-10 and, if so, what microbiological characteristics of KW3110 are important for phagocytosis by macrophages. Because IL-10 inhibits the production of IL-1β in activated macrophages [18,19], our results indicate that the strong suppression of IL-1β production by KW3110 might be achieved via the IL-10 signaling pathway.

RPE cells are damaged by various types of chronic stress. For example, blue light-induced RPE cell damage is one of the causes of retinal dysfunction, which possibly leads to eye fatigue [26,27]. Previous studies have shown that IL-10 produced by KW3110-stimulated macrophages suppresses RPE cell damage induced by blue light [10,11]. In addition, our study showed that KW3110 treatment suppressed prematurely senescent phenotypes in chronic inflammatory-stressed RPE cells (Figure 2, Figure 3 and Figure 4). The stress-induced senescence of RPE cells is caused by exposure to oxidants, and an accumulation of senescent RPE cells has been observed in the eyes of older monkeys [28,29,30]. In our study, KW3110 partially suppressed the aberrant expression of *claudin-1* in chronic inflammatory-stressed RPE cells (Figure 5). Although the immunofluorescence data are qualitative, KW3110 was shown to have possible effects on suppressing aberrant expressions of claudin-1 and ZO-1. RPE cells are essential for maintaining the blood–retina barrier, which is formed at the RPE cell layer and comprises tight junctions [22]. The expression of claudin-1 and ZO-1, key regulators of the function of tight junctions in epithelia, is upregulated and downregulated, respectively, in RPE cells after exposure to oxidants or pro-inflammatory cytokines [31,32,33]. Because tight junction molecules such as claudin-1 and ZO-1 interact to form the tight junction strands, the aberrant expression of claudin-1 and ZO-1 might cause a disruption of tight junctions in RPE cells [34]. Taken together, these findings enable us, for the first time, to propose a mechanism in which KW3110 might have a preventive effect on chronic inflammatory stress-induced RPE cell senescence and aberrant expression of tight junction molecules through the activation of macrophages and IL-10 signaling.

An increasing number of people are affected by chronic ocular disorders induced by the long-term use of display screens and/or mental stress. A previous clinical study showed that KW3110 ingestion ameliorates visual display terminal-induced eye fatigue [11]. Here, we used the Uchida–Kraepelin workload to investigate the effect of KW3110 on mental stress-induced ocular disorders. It has been reported that mental stress caused by continual calculation work, including the Uchida–Kraepelin test using visual display, might reduce CFF values and pupil reaction [13,14,35]; however, the Uchida–Kraepelin test might not be suitable for measuring mental stress [36]. Although both HFC-1 and miosis rate are established indices of eye condition, to our knowledge, no studies have investigated changes in these parameters due to the Uchida–Kraepelin workload [37,38]. Some of the present results suggest that the Uchida–Kraepelin test cannot induce ocular disorders related to mental stress (Table S4). Therefore, the supervising physician decided that only values recorded before the workload were suitable for the evaluation of the outcomes of chronic ocular disorder in the present study.

Changes in CFF values from the week-0 values were significantly higher throughout the ingestion period in the KW3110 group as compared with the placebo group (Figure 7 and Table 2). CFF reflects the excitation level of cerebral cortex via the visual system and is used as an index of eye fatigue related to mental fatigue [39,40]. A decrease in CFF is thought to indicate a reduction in the ability to process visual information [41]. A significant difference in CFF values between the two groups was detected before the workload, suggesting that the ingestion of KW3110 improves chronic ocular disorder. By contrast, there were no significant differences between the two groups either in HFC-1 values or miosis rates at any time points. HFC-1 values increase with the load on ciliary muscle, and miosis rates increase with the upregulation of adjustment function [42,43,44,45], suggesting that KW3110 ingestion has little effect on either ciliary muscle or adjustment function. Changes in the criteria “eye redness” and “unfocused vision” on the VAS from the week-0 value were significantly greater at weeks 4 and 8, respectively, in the KW3110 group as compared with the placebo group (Table S1). Note that the VAS is a subjective outcome and lower values represent a better condition [46]. Although focusing function is regulated mainly by the ciliary muscle, we did not observe any significant differences in HFC-1 values between the two groups [47]. In a previous study, KW3110 ingestion ameliorated the subjective symptom “eye redness” [11], a symptom that is thought to be worse in the case of hyperemia arising from sleep deprivation and allergic reaction caused by house dust [48]. In the POMS2 tests, “DD” values were significantly lower at week 4 and tended to remain lower throughout the ingestion period in the KW3110 group as compared with the placebo group (Table S6). Because a lower “DD” value represents a better mood state, our results suggest that ingestion of KW3110 may have a potential to suppress depression. Since “DD” value was evaluated as a secondary outcome in this study, further investigations focusing primarily on depression are required to conclude the effects of KW3110 on depression.

Regarding the safety endpoint evaluations, there were significant within-group and between-group differences in several parameters. Because the average values were all within the reference ranges [49], however, these differences were judged to be unrelated to the test supplements by the supervising physician. Therefore, continual ingestion of KW3110 for 8 weeks was indicated to be safe in this study.

In conclusion, our results demonstrate that KW3110 suppresses chronic inflammatory stress-induced RPE cell premature senescence and aberrant expression of tight junction molecules in vitro. In addition, KW3110 ingestion improved the objective parameter of eye fatigue among healthy humans. Furthermore, the continual ingestion of KW3110 for 8 weeks was found to be safe. Our findings reveal a protective effect of LAB on RPE cell senescence and chronic ocular disorders for the first time. Therefore, KW3110 might be a useful and safe tool to improve ocular disorders including eye fatigue.

## 4. Materials and Methods

### 4.1. In Vitro Experiments

#### 4.1.1. Materials

KW3110 was prepared as described previously [50]. Four strains of LAB other than KW3110 were prepared by isolation and culture from Japanese major commercial yogurt products, named strains A–D. LPS was purchased from Invivogen (San Diego, CA, USA). ATP was from Sigma (St. Louis, MO, USA). Hoechst 33342 and PI were from Dojindo (Tokyo, Japan). Rabbit anti-Claudin-1 antibody, rabbit anti-ZO-1 antibody, and goat anti-rabbit Immunoglobulin G (IgG) H&L (Alexa Fluor 488) were from Abcam (Cambridge, UK).

#### 4.1.2. Preparation of Human Monocytes

Human peripheral blood mononuclear cells from healthy donors were purchased from iQ Biosciences (Berkeley, CA, USA; catalog number: IQB-PBMC103). Monocytes were isolated from mononuclear cells by using a classical monocyte isolation kit (Miltenyi Biotec, Sunnyvale, CA, USA).

#### 4.1.3. Cell Culture

The mouse macrophage-like cell line (J774A.1), derived from ascites obtained from an adult female mouse with reticulum cell sarcoma [51] (ATCC (Manassas, VA, USA); catalog number: TIB-67), and human retinal pigment epithelium cell line (ARPE-19), derived from the normal eyes of a 19-year-old male [52] (ATCC (Manassas, VA, USA); catalog number: CRL-2302), were used. J774A.1 and ARPE-19 cells were maintained in, respectively, Dulbecco’s modified Eagle medium (DMEM) and DMEM:F-12 with 10% fetal bovine serum, 100 U/mL of penicillin, and 100 μg/mL of streptomycin (Gibco, Grand Island, NY, USA) at 37 °C in 5% CO_2_/air humidity. For the experiments involving supernatant transfer (Figure 2, Figure 3, Figure 4 and Figure 5), J774A.1 cells were seeded and incubated overnight in 24-well plates (1 × 10^5^ cells/well), and then treated with KW3110 for 24 h. The supernatant was collected and transferred to ARPE-19 cells, which had been seeded in 96-well plates (5 × 10^4^ cells/well), 24-well plates (1 × 10^5^ cells/well), or 35-mm dishes (2.5 × 10^4^ cells) 2 days before supernatant transfer. ARPE-19 cells were incubated for 0, 24, or 48 h before analysis. For the cell cycle assay, ARPE-19 cells were seeded in 24-well plates (5 × 10^5^ cells/well) with J774A.1 cell supernatant.

#### 4.1.4. Determination of Cytokine Production

Cells were seeded and incubated overnight in 24-well plates (J774A.1 cells, 1 × 10^5^ cells/well; human monocytes, 2.5 × 10^5^ cells/well), and then treated with KW3110 or strains A–D (5 μg/mL for J774A.1 cells and 10 μg/mL for human monocytes) for 24 h at 37 °C. For IL-1β measurement, J774A.1 cells were primed with 10 μg/mL of LPS for 4 h, and then stimulated with 2 mM ATP for 1 h. The supernatant was collected and centrifuged at 5000 rpm for 2 min. Cytokine levels were measured with a commercial enzyme-linked immunosorbent assay (ELISA) kit (mouse and human IL-10, BD Biosciences, San Jose, CA, USA; mouse IL-1β, eBioscience, San Diego, CA, USA).

#### 4.1.5. Cell Counts and Cell Death Analysis

ARPE-19 cells incubated for 0, 24, or 48 h after supernatant transfer in 96-well plates were stained with 1 μg/mL of Hoechst 33342 and PI solutions for 30 min at 37 °C. Hoechst 33342-positive cells were counted as the total cell number; the percentage of PI-positive cells in Hoechst 33342-positive cells was calculated as the dead cell ratio with an Operetta CLS system (PerkinElmer, Waltham, MA, USA).

#### 4.1.6. SPiDER-βGal Staining

ARPE-19 cells incubated for 24 h after supernatant transfer in 24-well plates were treated with Bafilomycin A1 solution for 1 h at 37 °C to suppress background signals. SPiDER-βGal solution containing Hoechst 33342 was added to the cells for 30 min at 37 °C. Bright-field and fluorescence images of the cells were obtained with a fluorescence microscope (KEYENCE, Osaka, Japan). The cell size in each bright-field image was measured with ImageJ software (National Institutes of Health, Bethesda, MD, USA). SPiDER-βGal-positive cells were counted in each fluorescence image and calculated as a ratio of the cells to Hoechst 33342-positive cells. All reagents used were from a Cellular Senescence Detection Kit – SPiDER-βGal (Dojindo, Tokyo, Japan).

#### 4.1.7. Quantitative Real-Time RT-PCR

Total RNA from ARPE-19 cells incubated for 24 h after supernatant transfer in 24-well plates was extracted with an RNeasy mini kit (QIAGEN, Venlo, Netherlands) and reverse-transcribed with a SuperScript IV First-Strand Synthesis System (Invitrogen, Waltham, MA, USA). Quantitative RT-PCR was performed with TB Green Premix Ex Taq (Takara Bio, Shiga, Japan) and a LightCycler 480 (Roche Diagnostics, Rotkreuz, Switzerland). The PCR conditions were as follows: 2-Step Cycling, 95 °C for 10 s hold; 45 cycles of 95 °C for 5 s and 60 °C for 20 s. All values were normalized to *GAPDH* expression. The specific forward and reverse primer pairs are listed in Table S13.

#### 4.1.8. Cell Cycle Assay

ARPE-19 cells incubated for 24 h after supernatant transfer in 24-well plates were treated with 1% Cell Cycle Assay Solution Deep Red (Dojindo, Tokyo, Japan) for 15 min at 37 °C to stain DNA. Flow cytometry analysis was performed on a FACS Canto II instrument and analyzed by using FlowJo software (BD, Franklin Lakes, NJ, USA).

#### 4.1.9. Immunocytochemistry

ARPE-19 cells incubated for 24 h after supernatant transfer in 35-mm dishes were fixed with 100% methanol (Wako, Osaka, Japan) for 5 min at room temperature, and incubated with PBS containing 10% goat serum (Wako, Osaka, Japan), 0.3 M glycine (Wako, Osaka, Japan), 1% bovine serum albumin (Wako, Osaka, Japan) and 0.1% tween (Bio-Rad Laboratories, Hercules, CA, USA) for 1 h at room temperature. The cells were then incubated with 1 μg/mL of rabbit anti-Claudin-1 or anti-ZO-1 antibody overnight at 4 °C, and with 1 μg/mL of goat anti-rabbit IgG H&L for 1 h at room temperature with 1 μg/mL of Hoechst 33342. Fluorescence images of the cells were obtained with a fluorescence microscope (KEYENCE, Osaka, Japan).

#### 4.1.10. Statistical Analysis

Values indicate means ± SEM. Statistical differences were analyzed by analysis of variance (ANOVA) followed by Tukey’s test. *p* values < 0.05 were considered statistically significant.

### 4.2. Clinical Study

#### 4.2.1. Ethics

This clinical study was approved by the Ethical Committee of the Takara Clinic, Medical Corporation Seishinkai (Tokyo, Japan) (1807-1806-KR01-01-TC, 30 July 2018) in accordance with the ethical standards established in the Helsinki Declaration and the ethical guidelines for epidemiological research of the Ministry of Education, Culture, Sports, Science and Technology, and the Ministry of Health, Labor and Welfare of Japan. Written informed consent was obtained from all subjects who received the appropriate information related to the study prior to enrollment. The study was registered with the University Hospital Medical Information Network in Japan Clinical Trials Registry (UMIN000033619, 3 August 2018) and was conducted in compliance with the protocol.

#### 4.2.2. Subjects

The subjects were healthy Japanese males and females of 35 to below 50 years with a complaint of eye fatigue. The inclusion criteria were: (1) healthy Japanese males and females of 35 to below 50 years, (2) individuals with complaint of eye fatigue, and (3) individuals with approval of admission to the study by the supervising physician. The exclusion criteria are listed in Table 3.

#### 4.2.3. Target Sample Size

The sample size was calculated by assuming that the effect size “d” of the intervention on any of the primary outcomes in this study was 0.65. Setting the significance level at 5% and the power at 80%, the number of subjects required per group was estimated to be 40. To account for an expected dropout rate of 10%, the number of subjects per group was fixed as 44.

#### 4.2.4. Test Supplements

We prepared two types of hard capsule as test supplements. Placebo supplements contained 200 mg of non-genetically engineered cornstarch. KW3110-containing supplements contained 50 mg of heat-killed KW3110 and 150 mg of non-genetically engineered cornstarch. The study controller confirmed that there were no discernible differences in appearance, taste, or smell between the two types of test supplements.

#### 4.2.5. Study Design

A randomized, double-blind, placebo-controlled parallel-group study was conducted by a contract research organization, ORTHOMEDICO Inc. (Tokyo, Japan), from July 2018 to April 2019 at the Ario Nishi-arai Eye Clinic (Tokyo, Japan). Subjects were screened for eligibility preceding the ingestion period based on the inclusion and exclusion criteria and interview by a supervising physician. The allocation manager randomly assigned enrolled subjects in a 1:1 ratio to the placebo group or the KW3110 group by stratified randomization with Statlight #11 Ver. 2.10 (Yukms, Kanagawa, Japan), considering (i) the difference in CFF value before and after the Uchida–Kraepelin workload at the screening test, (ii) an “ocular fatigue sensation” value on VAS before the Uchida–Kraepelin workload at the screening test, and (iii) sex and age. The allocation manager was independent from other organizations engaging this study and was not involved in determining subject eligibility, data collection, or analysis.

The manufacturer of test supplements printed a mark on the respective boxes enclosing the test supplements. The assignment list was kept secret by the allocation manager until database unlock. Throughout the study, the subjects, all investigators, and study personnel remained blinded. The subjects ingested one capsule of either the placebo or the KW3110-containing supplement per day after breakfast with water for 8 weeks. They were instructed to continue their usual lifestyle during the study and to visit the hospital for the test at screening, and weeks 0, 4, and 8. Compliance was monitored by interview and a diary kept by each subject.

Evaluations of eye condition were performed before and after the Uchida–Kraepelin workload, and after a 30-min rest period following the workload. The Uchida–Kraepelin workload was performed to evaluate mental stress-induced ocular disorder in accordance with the prescribed method [35].

#### 4.2.6. Primary Outcomes

The CFF, HFC-1, miosis rate, and VAS (eye conditions) were measured to evaluate the effect of KW3110 on ocular disorders induced by daily activities or mental stress. The CFF of the dominant eye was evaluated as an index of eye fatigue by using a Handy Flicker HF-II instrument (Neitz Instruments Co., Ltd., Tokyo, Japan) [39,40]. The HFC-1 and miosis rate of the dominant eye were evaluated as indices of eye-focusing function by using Auto Ref/Keratometer (NIDEK Co., Ltd., Aichi, Japan) and TriIRIS C9000 (Hamamatsu Photonics K.K., Shizuoka, Japan) instrument, respectively [14,53,54]. The VAS was used to evaluate how the subjects felt about their own eye conditions [46]. The criteria evaluated by the subjects using VAS are listed in Table S1. The data were obtained before and after the Uchida–Kraepelin workload and after the 30-min rest period at screening, and weeks 0, 4, and 8.

#### 4.2.7. Secondary Outcomes

Fatigue (by VAS), POMS2, and Uchida–Kraepelin tests were measured to evaluate the effect of KW3110 on other conditions. The VAS was used to evaluate how the subjects felt about their own fatigue condition [55]. POMS2 was used to evaluate the mood states of the subjects. The criteria evaluated by the subjects using POMS2 are listed in Table S6. The Uchida–Kraepelin tests were performed to evaluate subjects’ characteristics of attention and mental work ability [35]. The data for the VAS and POMS2 were obtained before and after the Uchida–Kraepelin workload and after the 30-min rest period at screening, and weeks 0, 4, or 8. Data for the Uchida–Kraepelin test were obtained at screening, and weeks 0, 4, and 8.

#### 4.2.8. Safety Endpoints

The circulatory analysis, urinalysis, peripheral blood analysis, ophthalmic analysis, and medical questionnaire were performed. All data were obtained before the Uchida–Kraepelin workload. The data for the circulatory analysis, urinalysis, and peripheral blood analysis were obtained before and after ingestion period. Nonspecific IgE was measured before ingestion period. The data for the ophthalmic analysis and medical questionnaire were obtained at screening, and weeks 0, 4, and 8.

#### 4.2.9. Statistical Analysis

Data analyses were performed by a statistical analyst in the contract research organization by using IBM SPSS Statistical software, version 23 (IBM, Armonk, NY, USA). Comparisons of interactions, main effects of ingestion, and each value between the two groups were performed by two-way repeated measures analysis of co-variance (ANCOVA) with the week-0 value as a covariate, followed by least squares difference (LSD) test. Comparisons of interactions, main effects of ingestion, and changes in each value from the week-0 value between the two groups were performed by two-way repeated measures ANOVA, followed by LSD test. Within-group comparisons between week 0 and week 4 or 8 were performed by ANOVA with subjects and times as fixed factors or repeated measures ANOVA, followed by LSD test. Comparisons of baseline characteristics of the subjects between the two groups were performed by χ^2^ test for the number of subjects (male/female) and dominant eye, and by unpaired Student’s *t*-test for other parameters (Table 1). *p* values < 0.05 were considered statistically significant.

## Figures and Tables

**Table 1:** Baseline characteristics of the study subjects.

Characteristic	KW3110	Placebo	*p* Values
	Mean ± SD	Mean ± SD	
Number of subjects (male/female)	40 (15/25)	40 (13/27)	0.6487
Age (years)	42.5 ± 3.9	42.4 ± 4.4	0.9150
Height (cm)	165.0 ± 7.6	161.7 ± 8.9	0.0787
Body weight (kg)	60.8 ± 10.2	59.5 ± 12.2	0.6059
BMI (kg/m^2^)	22.3 ± 2.9	22.7 ± 4.0	0.5604
Body fat ratio (%)	24.8 ± 6.1	25.9 ± 8.5	0.4973
Body temperature (°C)	36.3 ± 0.3	36.4 ± 0.3	0.5964
Nonspecific IgE (IU/mL)	105.1 ± 129.9	116.4 ± 177.9	0.7476
Dominant eye	Right: 27 Left: 13	Right: 26 Left: 14	1.0000

Data are expressed as means ± SD with the exception of sex and dominant eye. Comparisons between two groups were performed by using χ^2^ test for the number of subjects (male/female) and dominant eye, and by using unpaired Student’s *t*-test for other parameters.

**Table 2:** Effects of KW3110 on critical flicker frequency (CFF) values.

CFF (Hz)	KW3110			Placebo		
	Week 0	Week 4	Week 8	Week 0	Week 4	Week 8
	34.32 ± 1.79	35.51 ± 1.80 ^##^	34.41 ± 2.10	35.33 ± 3.14	35.65 ± 2.43	34.43 ± 2.88 ^#^
Change from the week-0 value ^$^		1.19 ± 1.40 *^,##^	0.09 ± 1.45 *		0.32 ± 2.24	−0.90 ± 2.54 ^#^

Data are expressed as means ± SD. Comparisons of change in CFF value from the week-0 value between the two groups were performed by two-way repeated measures ANOVA followed by least squares difference (LSD) test (^$^, *p* < 0.05; main effect of ingestion) (*, *p* < 0.05; effect of ingestion at each time point). Within-group comparisons between week 0 and week 4 or week 8 were performed by repeated measures ANOVA followed by LSD test (^#^, *p* < 0.05; ^##^, *p* < 0.01).

**Table 3:** Exclusion criteria.

1.	Subjects who were under treatment or had experienced malignancy, cardiac insufficiency, or heart attack
2.	Subjects who were under treatment or had experienced chronic diseases (e.g., arrhythmia, hepatopathy, nephropathy, cerebrovascular disorder, rheumatism, diabetes, dyslipidemia, hypertension)
3.	Subjects who were diagnosed with presbyopia or who were aware of having presbyopia previously
4.	Subjects with eye disease, entropion, trichiasis, or color blindness
5.	Subjects who were diagnosed with asthenopia
6.	Subjects who were taking eye drops for eye diseases
7.	Subjects with an uncorrected refractive error
8.	Subjects who had undergone laser in situ keratomileusis
9.	Subjects with severe astigmatism
10.	Subjects with amblyopia or strabismus
11.	Subjects with best-corrected visual acuity <1.0 for the dominant eye
12.	Subjects with a diagnosis of eye fatigue caused by something other than failed neurological function or ocular adjustment function
13.	Subjects who worked in a company developing or manufacturing foods with functional claims
14.	Subjects with excessive alcohol-drinking behavior
15.	Subjects who could not stop drinking alcoholic beverages for 2 days until the check-up
16.	Subjects who regularly took drugs or health foods with potential effects on the eyes or were expecting to use them during the study
17.	Subjects who could not stop taking drugs or health foods that might have effects on immune functions
18.	Subjects who could not stop eating foods similar to the test foods and/or were taking drugs or health foods including lactic acid bacteria or *Bifidobacterium*
19.	Subjects who had a tendency to get diarrhea after eating dairy products
20.	Subjects with the possibility of drug and/or food allergies
21.	Subjects with drug or alcohol dependence
22.	Subjects who were diagnosed with pollinosis
23.	Subjects who could not execute a work load test
24.	Subjects who were judged as unsuitable for the study from the background questionnaire
25.	Subjects who were pregnant, breastfeeding, or planning to get pregnant during the study
26.	Subjects who revoked the agreement/acquisition day and/or participated in other clinical studies within 3 months or were planning to participate in other clinical studies during this study
27.	Subjects who were judged as unsuitable for other reasons by the supervising physician

## Supplementary Materials

Supplementary materials can be found at https://www.mdpi.com/1422-0067/21/14/5091/s1.

## Abbreviations

ATP	adenosine 5′-triphosphate
CFF	critical flicker frequency
DD	depression-dejection
HFC-1	high frequency component
IL-1β	interleukin-1β
IL-10	interleukin-10
KW3110	*Lactobacillus paracasei* KW3110
LAB	lactic acid bacteria
LPS	ultrapure lipopolysaccharide from *E. coli* 0111:B4 strain
PI	propidium iodide
POMS2	profile of mood states 2nd edition
SASP	senescence-associated secretory phenotype
VAS	visual analogue scale
