# Supplementary material for: Lactobacillus paracasei KW3110 Suppresses Inflammatory Stress-Induced Premature Cellular Senescence of Human Retinal Pigment Epithelium Cells and Reduces Ocular Disorders in Healthy Humans
Source: Int J Mol Sci. 2020 Jul 18;21(14):5091. doi: 10.3390/ijms21145091 (PMC7403967; doi:10.3390/ijms21145091)
Supplement: Supplementary file 1 [file ijms-21-05091-s001.pdf]

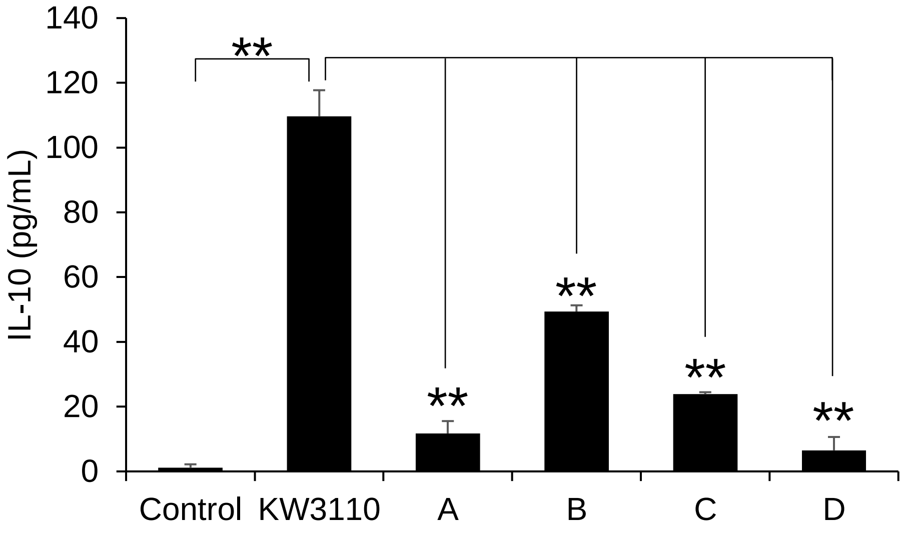

Figure S1. Comparative assay of the effects of LAB strains on IL-10 production in human monocytes. Human monocytes were treated with KW3110 or strains A-D (10 μg/mL) for 24 h and IL-10 levels in the supernatants were measured by ELISA. Values indicate means ± SEM. n = 3. Statistical differences were analyzed by ANOVA followed by Tukey's test (**, $P < 0.01$).

Table S1. Subjective symptoms of eye fatigue and related visual conditions.

Symptoms		KW3110			Placebo		
		week 0	week 4	week 8	week 0	week 4	week 8
Ocular fatigue sensation		48.33 ± 23.53	42.20 ± 21.04	40.45 ± 20.56 [#]	50.05 ± 23.26	44.90 ± 22.62	40.00 ± 21.46 [##]
	Change from week-0 value		-6.13 ± 22.69	-7.88 ± 23.40 [#]		-5.15 ± 18.11	-10.05 ± 19.80 [##]
Blurred vision		41.55 ± 22.28	36.25 ± 22.62	37.40 ± 21.94	46.98 ± 24.49	39.58 ± 25.00	36.60 ± 21.79 [##]
	Change from week-0 value		-5.30 ± 22.94	-4.15 ± 19.28		-7.40 ± 24.03	-10.38 ± 18.72 [##]
Ocular pain		29.03 ± 24.32	27.03 ± 21.86	28.85 ± 23.84	34.48 ± 25.13	28.73 ± 22.05	30.30 ± 23.29
	Change from week-0 value0		-2.00 ± 20.17	-0.18 ± 19.21		-5.75 ± 22.67	-4.18 ± 18.97
Dry eyes		46.00 ± 26.22	39.10 ± 22.71 [#]	37.23 ± 24.54 [##]	50.53 ± 25.02	41.68 ± 25.27 [##]	42.13 ± 22.30 [#]
	Change from week-0 value		-6.90 ± 20.23 [#]	-8.78 ± 19.78 [##]		-8.85 ± 20.63 [##]	-8.40 ± 20.88 [#]
Heavy eyes		36.80 ± 25.45	32.58 ± 23.09	34.93 ± 24.18	42.65 ± 24.28	38.10 ± 25.65	38.18 ± 24.92
	Change from week-0 value		-4.23 ± 24.25	-1.88 ± 22.41		-4.55 ± 21.36	-4.48 ± 22.24

Table S1. (continued)

Symptoms		KW3110 week 0	KW3110 week 4	KW3110 week 8	Placebo week 0	Placebo week 4	Placebo week 8
Sandy eyes		29.68 ± 23.37	26.65 ± 22.56	30.50 ± 25.10	36.65 ± 25.78	29.35 ± 23.15	30.08 ± 22.61
	Change from week-0 value		-3.03 ± 17.35	0.83 ± 18.92		-7.30 ± 23.27	-6.58 ± 27.06
Eye redness		28.45 ± 21.88	26.58 ± 21.56	29.30 ± 21.98	33.28 ± 21.16	23.83 ± 20.95 ##	24.10 ± 19.54 #
	Change from week-0 value $		-1.88 ± 20.34	0.85 ± 17.71 *		-9.45 ± 20.81 ##	-9.18 ± 22.30 #
Dazzled vision		27.35 ± 20.52	25.30 ± 20.40	28.23 ± 22.53	35.80 ± 24.16	27.15 ± 22.88 #	29.10 ± 21.34
	Change from week-0 value		-2.05 ± 16.54	0.88 ± 21.55		-8.65 ± 21.57 #	-6.70 ± 21.99
Double vision		28.38 ± 23.11	24.70 ± 20.28	27.13 ± 21.99	34.98 ± 23.99	28.20 ± 24.37	29.13 ± 22.68
	Change from week-0 value		-3.68 ± 17.97	-1.25 ± 19.94		-6.78 ± 21.89	-5.85 ± 22.30
Faded vision		32.90 ± 22.59	30.00 ± 21.59	32.28 ± 22.84	39.73 ± 25.90	29.40 ± 25.19 ##	30.18 ± 22.43 #
	Change from week-0 value $		-2.90 ± 18.44	-0.63 ± 18.10		-10.33 ± 21.21 ##	-9.55 ± 22.41 #

Table S1. (continued)

Symptoms		KW3110 week 0	KW3110 week 4	KW3110 week 8	Placebo week 0	Placebo week 4	Placebo week 8
Unfocused vision		28.70 ± 21.59	28.88 ± 22.39	30.20 ± 23.54	38.40 ± 26.54	29.28 ± 25.14 ##	30.85 ± 22.17 #
	Change from week-0 value $		0.18 ± 19.69 *	1.50 ± 21.07		-9.13 ± 21.24 ##	-7.55 ± 23.61 #
Stiffness of waist or shoulder		52.85 ± 25.65	44.63 ± 22.81 ##	43.53 ± 28.04 #	50.38 ± 22.64	41.83 ± 23.62 #	44.03 ± 22.06
	Change from week-0 value		-8.23 ± 16.09 ##	-9.33 ± 23.50 #		-8.55 ± 23.30 #	-6.35 ± 24.36
Stuffy head		33.93 ± 23.43	32.08 ± 22.43	31.98 ± 25.72	37.85 ± 22.46	32.43 ± 23.48	34.55 ± 21.91
	Change from week-0 value		-1.85 ± 14.43	-1.95 ± 22.72		-5.43 ± 21.47	-3.30 ± 25.88
Headache		28.30 ± 25.28	23.83 ± 22.71	25.23 ± 23.54	29.08 ± 22.92	25.83 ± 21.82	24.33 ± 20.87
	Change from week-0 value		-4.48 ± 18.96	-3.08 ± 19.29		-3.25 ± 18.81	-4.75 ± 20.04
Frustration		28.53 ± 24.70	26.23 ± 22.88	25.28 ± 23.08	32.13 ± 24.39	26.93 ± 21.45	25.23 ± 21.18
	Change from week-0 value		-2.30 ± 21.71	-3.25 ± 18.93		-5.20 ± 18.10	-6.90 ± 22.91

Table S1. (continued)

Data are expressed as mean ± SD. Comparisons of each value between two groups were performed by two-way repeated-measures ANCOVA with the week-0 value as a covariate. Comparisons of changes in value from the week-0 value between the two groups were performed by two-way repeated-measures ANOVA, followed by LSD test ($, $P < 0.05$; main effects of ingestion) (*, $P < 0.05$; effects of ingestion at each time point). Within-group comparisons between week 0 and week 4 or 8 were performed by repeated-measures ANOVA followed by LSD test ($^{\#}$, $P < 0.05$; $^{\#\#}$, $P < 0.01$).

Table S2. Effects of KW3110 on HFC-1 values.

HFC-1 (Hz)	KW3110			Placebo		
	week 0	week 4	week 8	week 0	week 4	week 8
	48.34 ± 5.41	48.45 ± 4.60	47.55 ± 5.00	48.07 ± 5.39	47.44 ± 5.63	47.08 ± 5.36
Change from week-0 value		0.11 ± 4.18	-0.78 ± 4.04		-0.63 ± 3.23	-0.99 ± 3.16

Data are expressed as mean ± SD. Comparisons of HFC-1 values between the two groups were performed by two-way repeated-measures ANCOVA with the week-0 value as a covariate. Comparisons of changes in HFC-1 values from the week-0 value between the two groups were performed by two-way repeated-measures ANOVA. Within-group comparisons between week 0 and week 4 or 8 were performed by repeated-measures ANOVA.

Table S3. Effects of KW3110 on miosis rates.

Miosis rate (%)	KW3110			Placebo		
	week 0	week 4	week 8	week 0	week 4	week 8
	35.66 ± 10.51	34.06 ± 12.52	34.55 ± 10.57	34.97 ± 11.74	37.17 ± 13.29	35.60 ± 11.07
Change from week-0 value		-1.60 ± 11.00	-1.12 ± 9.91		2.20 ± 13.00	0.63 ± 13.23

Data are expressed as mean ± SD. Comparisons of miosis rates between the two groups were performed by two-way repeated-measures ANCOVA with the week-0 value as a covariate. Comparisons of changes in miosis rates from the week-0 value between the two groups were performed by two-way repeated-measures ANOVA. Within-group comparisons between week 0 and week 4 or 8 were performed by repeated-measures ANOVA.

Table S4. Influence of Uchida–Kraepelin workload on CFF values, HFC-1 values, and miosis rates at week 0.

Parameters		KW3110	Placebo	*p* Values
		Mean ± SD	Mean ± SD	
CFF (Hz)	Before load	34.32 ± 1.79	35.33 ± 3.14	0.0814
	After load	33.71 ± 2.11	34.18 ± 3.58	0.4756
	After rest	33.48 ± 2.31	33.66 ± 3.35	0.7815
	Variation [a]	-0.61 ± 1.23	-1.15 ± 1.19	0.0498
	Variation [b]	-0.23 ± 1.27	-0.52 ± 1.44	0.3397
HFC-1 (Hz)	Before load	48.34 ± 5.41	48.07 ± 5.39	0.8267
	After load	48.97 ± 8.05	48.39 ± 4.80	0.6937
	After rest	47.70 ± 5.00	48.20 ± 4.86	0.6520
	Variation [a]	0.63 ± 6.78	0.31 ± 2.96	0.7847
	Variation [b]	-1.27 ± 6.91	-0.18 ± 4.20	0.3989
Miosis rate (%)	Before load	35.66 ± 10.51	34.97 ± 11.74	0.7812
	After load	33.06 ± 11.14	38.90 ± 11.20	0.0219
	After rest	33.13 ± 10.66	35.63 ± 11.85	0.3235
	Variation [a]	-2.61 ± 10.86	3.93 ± 10.27	0.0071
	Variation [b]	0.07 ± 8.82	-3.27 ± 10.76	0.1331

Data are expressed as mean ± SD. Comparisons between two groups were performed by unpaired Student's *t*-test. Before load, value recorded before Uchida–Kraepelin workload;, after load, value recorded after Uchida–Kraepelin workload; after rest, value recorded after rest; variation [a],

difference in each value between before and after Uchida–Kraepelin workload, variation [b], difference in each value between after Uchida–Kraepelin workload and after rest.

Table S5. Subjective symptom of fatigue.

Symptom	KW3110			Placebo		
	**week 0**	**week 4**	**week 8**	**week 0**	**week 4**	**week 8**
	44.20 ± 21.28	39.90 ± 18.67	40.23 ± 21.58	47.43 ± 15.75	46.25 ± 18.25	38.18 ± 17.77 ##
Change from week-0 value		-4.30 ± 25.63	-3.98 ± 25.73		-1.18 ± 17.37	-9.25 ± 16.77 ##

Data are expressed as mean ± SD. Comparisons of values between the two groups were performed by two-way repeated-measures ANCOVA with the week-0 value as a covariate. Comparisons of changes in value from the week-0 value between the two groups were performed by two-way repeated-measures ANOVA. Within-group comparisons between week 0 and week 4 or 8 were performed by repeated-measures ANOVA followed by LSD test (##, $P < 0.01$).

Table S6. Profile of mood states.

Parameters		KW3110 week 0	KW3110 week 4	KW3110 week 8	Placebo week 0	Placebo week 4	Placebo week 8
TMD		44.73 ± 6.80	42.53 ± 6.52 #	41.55 ± 5.64 ##	45.63 ± 6.78	45.38 ± 8.53	43.13 ± 6.77 #
	Change from week-0value		-2.20 ± 5.37 #	-3.18 ± 6.18 ##		-0.25 ± 8.59	-2.50 ± 5.86 #
AH		44.13 ± 5.64	42.70 ± 5.78	40.73 ± 4.27 ##	46.08 ± 7.89	45.28 ± 10.13	43.60 ± 8.58
	Change from week-0 value		-1.43 ± 6.02	-3.40 ± 6.22 ##		-0.80 ± 10.69	-2.48 ± 8.28
CB		44.08 ± 7.30	41.83 ± 6.53#	39.38 ± 6.28 ##	44.53 ± 7.28	43.08 ± 8.55	40.68 ± 8.16 ##
	Change from week-0value		-2.25 ± 7.24#	-4.70 ± 7.96 ##		-1.45 ± 6.58	-3.85 ± 5.76 ##
DD		45.75 ± 6.46	43.58 ± 4.78 *,##	42.65 ± 4.32 ##	46.08 ± 6.62	46.43 ± 8.00	44.43 ± 5.10
	Change from week-0 value $		-2.18 ± 4.76 ##	-3.10 ± 5.34 ##		0.35 ± 8.17	-1.65 ± 5.11
FI		47.08 ± 8.40	44.50 ± 9.14#	42.95 ± 7.99 ##	45.98 ± 6.83	45.35 ± 8.70	43.03 ± 7.86 #
	Change from week-0 value		-2.58 ± 8.34#	-4.13 ± 8.19 ##		-0.63 ± 9.45	-2.95 ± 8.95 #

Table S6. (continued)

Parameters		KW3110			Placebo		
		week 0	week 4	week 8	week 0	week 4	week 8
TA		46.85 ± 8.11	42.53 ± 7.55 $^{\#\#}$	42.38 ± 8.37 $^{\#\#}$	46.83 ± 9.03	45.40 ± 10.06	42.58 ± 9.45 $^{\#\#}$
	Change from week-0 value		-4.33 ± 7.49 $^{\#\#}$	-4.48 ± 8.12 $^{\#\#}$		-1.43 ± 9.54	-4.25 ± 7.18 $^{\#\#}$
VA		53.05 ± 10.29	51.43 ± 11.97	48.68 ± 10.94 $^{\#\#}$	50.70 ± 8.41	48.10 ± 9.95 $^{\#}$	47.95 ± 10.55 $^{\#}$
	Change from week-0 value		-1.63 ± 9.75	-4.38 ± 9.51 $^{\#\#}$		-2.60 ± 7.47 $^{\#}$	-2.75 ± 8.60 $^{\#}$
F		54.83 ± 10.75	51.60 ± 13.65 $^{\#}$	47.98 ± 13.91 $^{\#\#}$	50.93 ± 9.58	45.80 ± 11.87 $^{\#\#}$	44.60 ± 11.24 $^{\#\#}$
	Change from week-0 value		-3.23 ± 10.90 $^{\#}$	-6.85 ± 11.41 $^{\#\#}$		-5.13 ± 10.67 $^{\#\#}$	-6.33 ± 9.95 $^{\#\#}$

Data are expressed as mean ± SD. Comparisons of each value between the two groups were performed by two-way repeated-measures ANCOVA with the week-0 value as a covariate, followed by LSD test ($, $P < 0.05$; main effects of ingestion) (*, $P < 0.05$; effects of ingestion at each time point). Comparisons of changes in each value from the week-0 value between the two groups were performed by two-way repeated-measures ANOVA. Within-group comparisons between week 0 and week 4 or 8 were performed by ANOVA with subjects and times as fixed factors followed by LSD test ($^{\#}$, $P < 0.05$; $^{\#\#}$, $P < 0.01$). TMD, total mood disturbance; TA, tension-anxiety; DD, depression–dejection; AH, anger-hostility; VA, vigor-activity; FI, fatigue-interia; CB, confusion-bewilderment; F, friendliness.

Table S7. Performance during Uchida–Kraepelin workload.

Parameters		KW3110			Placebo		
		week 0	week 4	week 8	week 0	week 4	week 8
Total workload		114.48 ± 31.96	120.32 ± 33.12 [##]	119.94 ± 36.95 [#]	107.76 ± 29.79	111.00 ± 28.08	111.02 ± 29.21
	Change from week-0 value		5.84 ± 9.12 [##]	5.46 ± 12.93 [#]		3.24 ± 13.35	3.26 ± 13.38
Average workload		57.24 ± 15.98	60.16 ± 16.56 [##]	59.97 ± 18.48 [#]	53.88 ± 14.90	55.50 ± 14.04	55.51 ± 14.60
	Change from week-0 value		2.92 ± 4.56 [##]	2.73 ± 6.47 [#]		1.62 ± 6.68	1.63 ± 6.69
Primacy effect rate		1.12 ± 0.09	1.11 ± 0.11	1.13 ± 0.12	1.11 ± 0.09	1.11 ± 0.08	1.11 ± 0.09
	Change from week-0 value		-0.01 ± 0.09	0.01 ± 0.09		0.00 ± 0.11	0.00 ± 0.10
Upset rate		0.33 ± 0.14	0.34 ± 0.16	0.35 ± 0.20	0.30 ± 0.09	0.34 ± 0.16	0.35 ± 0.17 [#]
	Change from week-0 value		0.01 ± 0.11	0.02 ± 0.16		0.04 ± 0.16	0.05 ± 0.15 [#]
Correct answer rate		0.99 ± 0.01	0.99 ± 0.01	0.99 ± 0.01	0.99 ± 0.02	0.99 ± 0.01 [#]	0.99 ± 0.01
	Change from week-0 value		0.00 ± 0.01	0.00 ± 0.01		0.00 ± 0.01 [#]	0.00 ± 0.02

Table S7. (continued)

Parameters		KW3110 week 0	KW3110 week 4	KW3110 week 8	Placebo week 0	Placebo week 4	Placebo week 8
Total correct answer rate		0.99 ± 0.01	0.99 ± 0.01	0.99 ± 0.01	0.99 ± 0.02	0.99 ± 0.01 #	0.99 ± 0.01
	Change from week-0 value		0.00 ± 0.01	0.00 ± 0.01		0.00 ± 0.01 #	0.00 ± 0.02
Break effect rate		1.07 ± 0.05	1.04 ± 0.06 #	1.04 ± 0.08	1.08 ± 0.06	1.12 ± 0.33	1.05 ± 0.10
	Change from week-0 value		-0.03 ± 0.07 #	-0.03 ± 0.10		0.04 ± 0.33	-0.03 ± 0.10
Break growth rate		0.07 ± 0.05	0.04 ± 0.06 #	0.04 ± 0.08	0.08 ± 0.06	0.12 ± 0.33	0.05 ± 0.10
	Change from week-0 value		-0.03 ± 0.07 #	-0.03 ± 0.10		0.04 ± 0.33	-0.03 ± 0.10

Data are expressed as mean ± SD. Comparisons of each value between the two groups were performed by two-way repeated-measures ANCOVA with the week-0 value as a covariate. Comparisons of changes in each value from the week-0 value between the two groups were performed by two-way repeated-measures ANOVA. Within-group comparisons between week 0 and week 4 or 8 were performed by repeated-measures ANOVA followed by LSD test (#, $P < 0.05$; ##, $P < 0.01$).

Table S8. Circulatory parameters.

Parameters		KW3110 before ingestion period	KW3110 after ingestion period	Placebo before ingestion period	Placebo after ingestion period
SBP (mmHg)		112.73 ± 9.90	115.02 ± 11.12	113.52 ± 15.62	115.91 ± 14.20
	Change from value at Scr		2.29 ± 9.59		2.38 ± 8.26
DBP (mmHg)		72.19 ± 8.36	74.05 ± 9.00	72.73 ± 11.99	73.83 ± 11.14
	Change from value at Scr		1.86 ± 7.60		1.09 ± 6.09
Pulse rate (bpm)		70.10 ± 9.58 **	71.23 ± 9.58	76.05 ± 11.12	77.65 ± 13.31
	Change from value at Scr		1.13 ± 8.60		1.60 ± 13.30

Data are expressed as mean ± SD. Comparisons of each value after the ingestion period between the two groups were performed by two-way repeated-measures ANCOVA with the value before the ingestion period as a covariate. Comparisons of each value before the ingestion period and changes in each value from the value before the ingestion period between the two groups were performed by unpaired Student's *t*-test (**, *P* < 0.01). Within-group comparisons between the values recorded before and after the ingestion period were performed by paired Student's *t*-test. Scr, screening.

Table S9. Urinalysis.

Parameters	Reference range	KW3110				Placebo			
		before Ingestion period		after ingestion period		before Ingestion period		after ingestion period	
		In	Out	In	Out	In	Out	In	Out
Protein	-	42	1	38	5	37	6	41	2
Glucose	-	43	0	43	0	43	0	42	1
Urobilinogen	±	43	0	43	0	43	0	41	2
Bilirubin	-	43	0	43	0	43	0	43	0
pH	5.0-7.5	41	2	42	1	41	2	43	0
Occult blood	-	39	4	39	4	34	9	37	6
Ketone body	-	43	0	43	0	42	1	43	0

Data are expressed as the number of subjects whose parameters were inside or outside the reference ranges. Comparisons between the two groups were performed by χ^2 test. Within-group comparisons between values recorded before and after the ingestion period were performed by McNemar test. In, inside reference range; Out, outside reference range.

Table S10. Peripheral blood analysis.

Parameters	Reference range		KW3110 before Ingestion period	KW3110 after ingestion period	Placebo before Ingestion period	Placebo after ingestion period
WBC (/μL)	3300-9000		5825.58 ± 1457.18	5695.35 ± 1148.70	5581.40 ± 1269.09	5846.51 ± 1285.51
		Change from value at Scr		-130.23 ± 1162.41		265.12 ± 1141.40
RBC (×10^4/μL)	Male: 430-570<br>Female: 380-500		460.72 ± 48.59	464.07 ± 45.14	447.14 ± 38.73	448.60 ± 40.30
		Change from value at Scr		3.35 ± 22.96		1.47 ± 17.14
Hb (g/dL)	Male: 13.5-17.5<br>Female: 11.5-15.0		13.69 ± 1.80	13.86 ± 1.72	13.33 ± 1.62	13.41 ± 1.67
		Change from value at Scr		0.17 ± 0.62		0.08 ± 0.71
Ht (%)	Male: 39.7-52.4<br>Female: 34.8-45.0		42.57 ± 4.33	42.89 ± 4.05	41.50 ± 3.97	41.51 ± 4.14
		Change from value at Scr		0.32 ± 1.81		0.01 ± 1.80
Plt (×10^4/μL)	14.0-34.0		27.95 ± 6.55	28.15 ± 6.85	30.75 ± 6.55	30.60 ± 6.80
		Change from value at Scr		0.20 ± 3.10		-0.15 ± 2.87

Table S10. (continued)

Parameters	Reference range		KW3110 before Ingestion period	KW3110 after ingestion period	Placebo before Ingestion period	Placebo after ingestion period
MCV (fL)	85-102		92.56 ± 5.56	92.65 ± 5.11	92.88 ± 4.86	92.63 ± 5.06
		Change from value at Scr		0.09 ± 2.17		-0.26 ± 2.41
MCH (pg)	28.0-34.0		29.73 ± 2.37	29.87 ± 2.36	29.79 ± 2.35	29.87 ± 2.28
		Change from value at Scr		0.14 ± 0.70		0.09 ± 1.14
MCHC (%)	30.2-35.1		32.08 ± 1.24	32.24 ± 1.25	32.04 ± 1.25	32.24 ± 1.13
		Change from value at Scr		0.16 ± 0.64		0.20 ± 0.87
Neutrophil rate (%)	40.0-75.0		61.04 ± 8.31	60.74 ± 7.27	58.95 ± 7.68	60.10 ± 8.43
		Change from value at Scr		-0.30 ± 6.26		1.15 ± 6.78
Lymphocyte rate (%)	18.0-49.0		30.62 ± 7.48	30.46 ± 6.09	32.79 ± 7.16	31.38 ± 7.99
		Change from value at Scr		-0.16 ± 5.91		-1.40 ± 6.10

Table S10. (continued)

Parameters	Reference range		KW3110 before Ingestion period	KW3110 after ingestion period	Placebo before Ingestion period	Placebo after ingestion period
Monocyte rate (%)	2.0-10.0		5.26 ± 1.42	5.21 ± 1.13	4.73 ± 1.14	4.78 ± 1.21
		Change from value at Scr		-0.05 ± 1.14		0.05 ± 1.17
Eosinophil rate (%)	0.0-8.0		2.43 ± 2.35	2.84 ± 2.28	2.82 ± 2.01	3.07 ± 2.46
		Change from value at Scr		0.41 ± 2.42		0.25 ± 1.63
Basophil rate (%)	0.0-2.0		0.64 ± 0.50	0.75 ± 0.45	0.71 ± 0.46	0.67 ± 0.49
		Change from value at Scr		0.10 ± 0.37		-0.04 ± 0.38
Neutrophils (/μL)	-		3633.81 ± 1299.09	3502.98 ± 986.83	3333.25 ± 1047.81	3566.33 ± 1089.72
		Change from value at Scr		-130.83 ± 978.72		233.08 ± 1012.05
Lymphocytes (/μL)	-		1712.99 ± 329.63	1692.58 ± 321.12	1791.45 ± 453.90	1789.20 ± 481.25
		Change from value at Scr		-20.41 ± 342.70		-2.25 ± 282.40

Table S10. (continued)

Parameters	Reference range		KW3110 before Ingestion period	KW3110 after ingestion period	Placebo before Ingestion period	Placebo after ingestion period
Monocytes (/μL)	-		303.39 ± 104.52	295.78 ± 83.12	263.33 ± 81.69	275.02 ± 83.43
		Change from the value at Scr		-7.60 ± 71.63		11.69 ± 87.77
Eosinophils (/μL)	-		139.90 ± 135.12	161.33 ± 133.57	155.50 ± 115.01	178.36 ± 150.22
		Change from value at Scr		21.43 ± 139.96		22.86 ± 90.77
Basophils (/μL)	-		35.50 ± 25.40	42.67 ± 25.19 [#]	37.86 ± 22.47	37.60 ± 26.82
		Change from value at Scr		7.17 ± 20.75 [#]		-0.26 ± 18.49
AST (U/L)	10-40		20.09 ± 4.82	20.28 ± 6.29	19.58 ± 5.27	18.98 ± 6.40
		Change from value at Scr		0.19 ± 3.92		-0.60 ± 5.07
ALT (U/L)	5-45		17.09 ± 6.83	17.40 ± 8.75	18.40 ± 10.78	16.95 ± 9.90
		Change from value at Scr		0.30 ± 5.61		-1.44 ± 7.40

Table S10. (continued)

Parameters	Reference range		KW3110 before Ingestion period	KW3110 after ingestion period	Placebo before Ingestion period	Placebo after ingestion period
γ-GTP (U/L)	Male: ≤80<br>Female: ≤30		28.40 ± 23.13	29.02 ± 23.79	21.67 ± 13.32	21.05 ± 12.57
		Change from value at Scr		0.63 ± 13.08		-0.63 ± 5.80
ALP (U/L)	100-325		173.37 ± 44.40	174.84 ± 61.10	180.72 ± 63.69	175.77 ± 59.42
		Change from value at Scr		1.47 ± 28.72		-4.95 ± 20.40
LDH (U/L)	120-240		170.26 ± 23.97	170.12 ± 25.08	173.28 ± 24.49	175.28 ± 24.25
		Change from value at Scr		-0.14 ± 10.28		2.00 ± 16.77
LAP (U/L)	Male: 45-81<br>Female: 37-61		49.16 ± 11.32	49.63 ± 11.17	49.33 ± 6.77	49.14 ± 7.41
		Change from value at Scr		0.47 ± 4.51		-0.19 ± 3.69
Total bilirubin (mg/dL)	0.2-1.2		0.86 ± 0.31	0.84 ± 0.27	0.80 ± 0.30	0.78 ± 0.27
		Change from value at Scr		-0.02 ± 0.29		-0.02 ± 0.26

Table S10. (continued)

Parameters	Reference range		KW3110 before Ingestion period	KW3110 after ingestion period	Placebo before Ingestion period	Placebo after ingestion period
Direct bilirubin (mg/dL)	0.0-0.2		0.07 ± 0.05	0.07 ± 0.05	0.06 ± 0.05	0.07 ± 0.05
		Change from value at Scr		0.00 ± 0.05		0.01 ± 0.06
Indirect bilirubin (mg/dL)	0.2-1.0		0.79 ± 0.28	0.77 ± 0.24	0.74 ± 0.26	0.71 ± 0.25
		Change from value at Scr		-0.01 ± 0.27		-0.03 ± 0.23
Cholinesterase (U/L)	Male: 234-493 Female: 200-452		314.23 ± 69.36	316.30 ± 74.74	298.70 ± 65.30	301.79 ± 65.42
		Change from value at Scr		2.07 ± 21.09		3.09 ± 19.27
Total protein (g/dL)	6.7-8.3		6.97 ± 0.43	7.10 ± 0.34 [#]	7.02 ± 0.44	7.08 ± 0.37
		Change from value at Scr		0.13 ± 0.35 [#]		0.06 ± 0.34
Urea nitrogen (mg/dL)	8.0-20.0		12.52 ± 3.09	12.71 ± 2.99	12.33 ± 2.69	12.60 ± 2.61
		Change from value at Scr		0.19 ± 2.74		0.27 ± 2.36

Table S10. (continued)

Parameters	Reference range		KW3110 before Ingestion period	KW3110 after ingestion period	Placebo before Ingestion period	Placebo after ingestion period
Creatinine (mg/dL)	Male: 0.61-1.04<br>Female: 0.47-0.79		0.70 ± 0.14	0.69 ± 0.14	0.65 ± 0.12	0.65 ± 0.13
		Change from value at Scr		-0.01 ± 0.05		0.00 ± 0.05
Uric acid (mg/dL)	Male: 3.8-7.0<br>Female: 2.5-7.0		5.04 ± 1.34 *	4.86 ± 1.24	4.46 ± 1.31	4.40 ± 1.33
		Change from value at Scr		-0.18 ± 0.59		-0.06 ± 0.52
CK (U/L)	Male: 60-270<br>Female: 40-150		103.40 ± 56.42	108.95 ± 60.69	108.14 ± 63.92	107.14 ± 53.81
		Change from value at Scr		5.56 ± 49.40		-1.00 ± 48.77
Na (mEq/L)	137-147		139.95 ± 1.91	139.84 ± 2.02	139.95 ± 1.50	139.91 ± 1.38
		Change from value at Scr		-0.12 ± 1.43		-0.05 ± 1.51
K (mEq/L)	3.5-5.0		4.43 ± 0.45	4.37 ± 0.34	4.30 ± 0.38	4.31 ± 0.33
		Change from value at Scr		-0.06 ± 0.45		0.00 ± 0.37

Table S10. (continued)

Parameters	Reference range		KW3110 before Ingestion period	KW3110 after ingestion period	Placebo before Ingestion period	Placebo after ingestion period
Cl (mEq/L)	98-108		101.47 ± 1.68	101.72 ± 1.76	101.74 ± 1.60	101.93 ± 1.71
		Change from value at Scr		0.26 ± 1.63		0.19 ± 1.78
Ca (mg/dL)	8.4-10.4		9.09 ± 0.36	9.11 ± 0.39	9.06 ± 0.26	9.06 ± 0.37
		Change from value at Scr		0.02 ± 0.34		0.00 ± 0.34
Inorganic phosphorus (mg/dL)	2.5-4.5		3.27 ± 0.57	3.14 ± 0.50	3.27 ± 0.58	3.28 ± 0.44
		Change from value at Scr		-0.13 ± 0.55		0.01 ± 0.49
Fe (μg/dL)	Male: 50-200<br>Female: 40-180		100.23 ± 55.63	98.05 ± 41.99	94.84 ± 42.05	96.86 ± 44.80
		Change from value at Scr		-2.19 ± 51.95		2.02 ± 42.44
Amylase (U/L)	40-122		80.44 ± 24.11	80.56 ± 23.88	78.67 ± 22.46	77.51 ± 20.83
		Change from value at Scr		0.12 ± 12.89		-1.16 ± 11.41

Table S10. (continued)

Parameters	Reference range		KW3110 before Ingestion period	KW3110 after ingestion period	Placebo before Ingestion period	Placebo after ingestion period
Total cholesterol (mg/dL)	120-219		201.72 ± 31.97	205.67 ± 39.37	210.93 ± 35.55	214.44 ± 36.52
		Change from value at Scr		3.95 ± 19.59		3.51 ± 17.11
HDL-cholesterol (mg/dL)	Male: 40-85<br>Female: 40-95		67.05 ± 17.64	66.86 ± 18.20	69.56 ± 18.65	71.53 ± 19.32
		Change from value at Scr		-0.19 ± 4.78		1.98 ± 6.69
LDL-cholesterol (mg/dL)	65-139		117.26 ± 29.33	120.91 ± 34.66	124.70 ± 32.13	127.26 ± 35.88
		Change from value at Scr		3.65 ± 15.59		2.56 ± 14.89
TG (mg/dL)	30-149		96.23 ± 71.46	101.44 ± 69.54	89.26 ± 78.89	86.47 ± 49.73
		Change from value at Scr		5.21 ± 37.58		-2.79 ± 60.92
Glucose (mg/dL)	70-109		81.35 ± 6.55	82.77 ± 7.98	83.44 ± 7.58	85.05 ± 8.23
		Change from value at Scr		1.42 ± 5.93		1.60 ± 8.50

Table S10. (continued)

Parameters	Reference range		KW3110		Placebo	
			before Ingestion period	after ingestion period	before Ingestion period	after ingestion period
HbA1c (%)	4.6-6.2		5.24 ± 0.22	5.27 ± 0.21	5.30 ± 0.26	5.33 ± 0.26
		Change from value at Scr		0.03 ± 0.21		0.04 ± 0.17
Glycoalbumin (%)	12.3-16.5		13.71 ± 1.13	13.79 ± 1.26	13.94 ± 1.24	13.93 ± 1.12
		Change from value at Scr		0.08 ± 0.60		-0.01 ± 0.68

Data are expressed as mean ± SD. Comparisons of each value after the ingestion period between the two groups were performed by two-way repeated-measures ANCOVA with the value before the ingestion period as a covariate. Comparisons of each value before the ingestion period and changes in each value from the value before the ingestion period between the two groups were performed by unpaired Student's *t*-test (*, $P < 0.05$). Within-group comparisons between the values recorded before and after the ingestion period were performed by paired Student's *t*-test (#, $P < 0.05$).

Table S11. Ophthalmic analysis.

Parameter		KW3110				Placebo			
		Scr	week 0	week 4	week 8	Scr	week 0	week 4	week 8
Eyesight (average of both eyes)		0.61 ± 0.54	0.55 ± 0.51	0.59 ± 0.57	0.63 ± 0.58	0.57 ± 0.53	0.54 ± 0.52	0.54 ± 0.49	0.52 ± 0.48
	Change from week-0 value			-0.02 ± 0.27	0.02 ± 0.43			-0.03 ± 0.20	-0.05 ± 0.20
Eyesight (dominant eye)		0.61 ± 0.53	0.55 ± 0.51	0.60 ± 0.59	0.68 ± 0.84	0.57 ± 0.53	0.56 ± 0.52	0.53 ± 0.49	0.51 ± 0.48
	Change from week-0 value			-0.01 ± 0.32	0.07 ± 0.78			-0.05 ± 0.24	-0.07 ± 0.22
Eyesight (non-dominant eye)		0.61 ± 0.58	0.56 ± 0.53	0.58 ± 0.57	0.58 ± 0.54	0.57 ± 0.56	0.53 ± 0.54	0.55 ± 0.51	0.53 ± 0.50
	Change from week-0 value			-0.04 ± 0.26	-0.03 ± 0.24			-0.02 ± 0.22	-0.04 ± 0.25
Eyesight (right eye)		0.63 ± 0.55	0.55 ± 0.52	0.62 ± 0.61	0.59 ± 0.53	0.56 ± 0.52	0.54 ± 0.51	0.52 ± 0.48	0.51 ± 0.47
	Change from week-0 value			-0.01 ± 0.29	-0.04 ± 0.20			-0.04 ± 0.24	-0.05 ± 0.20

Table S11. (continued)

Parameter		KW3110 Scr			KW3110 week 0			KW3110 week 4			KW3110 week 8			Placebo Scr			Placebo week 0			Placebo week 4			Placebo week 8		
Eyesight (left eye)		0.60	±	0.56	0.55	±	0.52	0.56	±	0.56	0.67	±	0.84	0.59	±	0.56	0.54	±	0.55	0.57	±	0.52	0.53	±	0.50
	Change from week-0 value							-0.04	±	0.29	0.08	±	0.79							-0.02	±	0.22	-0.06	±	0.26
Intraocular pressure (average of both eyes) (mmHg)	$	13.93	±	2.44	13.86	±	2.38	13.44	±	2.88 *	13.97	±	2.75	14.67	±	2.73	14.44	±	2.79	14.94	±	2.76	15.06	±	3.10
	Change from week-0 value $							-0.49	±	1.98	0.03	±	2.07							0.26	±	1.84	0.39	±	1.89
Intraocular pressure (dominant eye) (mmHg)	$$	13.89	±	2.84	14.21	±	3.16	13.47	±	3.50	14.07	±	3.25	14.71	±	2.97	14.35	±	2.99	15.05	±	2.97	15.26	±	3.71
	Change from week-0 value $$							-0.43	±	2.47	0.18	±	2.36							0.34	±	2.15	0.54	±	2.48
Intraocular pressure (non-dominant eye) (mmHg)		13.98	±	2.49	13.51	±	2.39	13.42	±	2.74 *	13.86	±	2.58	14.63	±	2.73	14.53	±	2.84	14.82	±	2.99	14.87	±	2.80
	Change from week-0 value							-0.56	±	2.06	-0.12	±	2.28							0.19	±	2.27	0.23	±	2.10

Table S11. (continued)

Parameter		KW3110				Placebo			
		Scr	week 0	week 4	week 8	Scr	week 0	week 4	week 8
Intraocular pressure (right eye) (mmHg)	$$	13.76 ± 2.58	14.26 ± 2.75	13.26 ± 2.81 *	14.00 ± 2.58	14.77 ± 2.88	14.40 ± 2.95	14.98 ± 3.08	14.81 ± 3.22
	Change from week-0 value $$			-0.50 ± 2.16	0.24 ± 2.30			0.22 ± 2.25	0.05 ± 2.15
Intraocular pressure (left eye) (mmHg)		14.11 ± 2.76	13.47 ± 2.84	13.63 ± 3.43	13.93 ± 3.25	14.58 ± 2.82	14.49 ± 2.89	14.89 ± 2.88	15.31 ± 3.34 #
	Change from week-0 value			-0.48 ± 2.39	-0.18 ± 2.33			0.31 ± 2.17	0.73 ± 2.39 #

Data are expressed as mean ± SD. Comparisons of each value between the two groups were performed by two-way repeated-measures ANCOVA with the week-0 value as a covariate, followed by LSD test ($, $P < 0.05$; main effects of ingestion) (*, $P < 0.05$; effects of ingestion at each time point). Comparisons in changes of each value from the week-0 value between the two groups were performed by two-way repeated-measures ANOVA. Within-group comparisons between week 0 and week 4 or 8 were performed by repeated-measures ANOVA followed by LSD test (#, $P < 0.05$).

Table S12. Medical questionnaire.

Symptoms	KW3110						Placebo					
	week 0		week 4		week 8		week 0		week 4		week 8	
	Yes	No	Yes	No	Yes	No	Yes	No	Yes	No	Yes	No
Poor health	2	41	1	42	0	43	3	40	0	43	4	39
Palpitation	0	43	0	43	0	43	0	43	0	43	0	43
Lassitude	1	42	0	43	0	43	2	41	0	43	0	43
Skin problems	0	43	0	43	0	43	0	43	0	43	1	42
Anorexia	0	43	0	43	0	43	0	43	0	43	0	43
Stomachache	0	43	0	43	0	43	0	43	0	43	0	43
Diarrhea	0	43	0	43	0	43	0	43	0	43	0	43
Constipation	1	42	0	43	0	43	1	42	0	43	2	41
Headache	1	42	0	43	0	43	0	43	1	42	1	42
Dizzy	0	43	0	43	0	43	0	43	0	43	0	43
Nausea	0	43	0	43	0	43	0	43	0	43	0	43
Swelling	1	42	0	43	0	43	0	43	0	43	1	42
Sweaty	0	43	0	43	0	43	0	43	0	43	0	43

Table S12. (continued)

Symptoms	KW3110						Placebo					
	week 0		week 4		week 8		week 0		week 4		week 8	
	Yes	No	Yes	No	Yes	No	Yes	No	Yes	No	Yes	No
Pollakiuria	0	43	0	43	0	43	1	42	0	43	0	43
Hot flashes	0	43	0	43	0	43	0	43	0	43	0	43
Sleep deprivation	1	42	0	43	0	43	1	42	0	43	1	42
Dry mouth	0	43	0	43	0	43	0	43	0	43	0	43
Others	0	43	1	42	0	43	1	42	0	43	1	42

Data are expressed as the number of subjects who answered "Yes" or "No" to each question. The comparisons between two groups were performed by a χ^2 test. The within-group comparisons between week 0 and week 4 or 8 were performed by a McNemar test.

Table S13. Primer sequences used for quantitative real-time RT-PCR.

Gene	Forward primer (5’-3’)	Reverse primer (5’-3’)
GAPDH	AGGCTAGCTGGCCCGATTTC	TGGCAACAATATCCACTTTACCAGA
p53	CATCCTCACCATCATCACAC	CTCCATCCAGTGGTTTCTTC
p21	CGATGGAACTTCGACTTTGT	AGGCACAAGGGTACAAGA
IL-6	CTCCAGAACAGATTTGAGAGTAG	GCGCAGAATGAGATGAGTT
IL-8	CTCTTGGCAGCCTTCCT	AGACAGAGCTCTCTTCCATC
IL-1β	CTGTCCTGCGTGTTGAAAGATG	TTCTGCTTGAGAGGTGCTGATG
ZO-1	GAAGCTGATGGTGTGGATAG	GTCTGGTTTGGACACTAAGG
claudin-1	GATGAGGTGCAGAAGATGAG	GGACAGGAACAGCAAAGTAG
